# Supplementary material for: Basal Diet Determined Long-Term Composition of the Gut Microbiome and Mouse Phenotype to a Greater Extent than Fecal Microbiome Transfer from Lean or Obese Human Donors
Source: Nutrients. 2019 Jul 17;11(7):1630. doi: 10.3390/nu11071630 (PMC6682898; doi:10.3390/nu11071630)
Supplement: Supplementary file 1 [file nutrients-11-01630-s001.pdf]

nutrients

MDPI

Basal diet determined long-term composition of the gut microbiome and mouse phenotype to a greater extent than fecal microbiome transfer from lean or obese human donors

Daphne M. Rodriguez[1], Abby D. Benninghoff[1,3]*, Niklas D.J. Aardema[2], Sumira Phatak[1], and Korry J. Hintze[2,3]*

1. Department of Animal, Dairy and Veterinary Sciences, Utah State University, Logan, UT 84322, USA
2. Department of Nutrition, Dietetics and Food Sciences, Utah State University, Logan, UT 84322, USA
3. USTAR Applied Nutrition Research, Utah State University, Logan, UT 84322, USA

* **Corresponding author:** Abby D. Benninghoff, Utah State University, Department of Animal, Dairy and Veterinary Sciences, 4815 Old Main Hill, Logan, UT 84322-4815, USA, Phone: 435-797-8649, Email: abby.benninghoff@usu.edu, Korry J. Hintze, Utah State University, Department of Nutrition, Dietetics and Food Sciences, 8700 Old Main Hill, Logan, UT 84322-8700, USA, Phone: 435-797-2124, Email: korry.hintze@usu.edu

SUPPLEMENTAL MATERIALS

Table S1 Parameters of donors

Donor	Age	BMI	Waist circumference (cm)	Ethnicity	Sex
Lean					
L1	65	22.5	89.2	White	Male
L2	32	20.1	82.7	White	Male
L3	29	22.6	75.0	Asian	Male
Obese					
O4	28	34.8	113.7	White	Male
O5	28	30.6	101.5	White	Male
O6	65	31.0	109.5	White	Male

Abbreviations: *BMI* body mass index

Table S2 Composition of experimental diets

Nutrient	AIN93G	DIO	TWD
Energy density (kcal/g)	3.8	4.6	4.4
Macronutrient			
Carbohydrates (g/kg diet)			
Corn starch	398	85	230
Maltodextrin	132	115	70
Sucrose	100	200	261
Cellulose	50	58	30
kcal (% of total)	*60.1%*	*36.2%*	*54.5%*
Proteins (g/kg)			
Casein	200	245	190
L-cystine	3	3.5	2.8
kcal (% of total)	*18.8%*	*19.0%*	*15.4%*
Fats (g/kg)			
Soybean oil	70	30	31.4
Anhydrous milk fat			36.3
Olive oil			28.0
Lard		195	28.0
Beef tallow			24.8
Corn oil			16.5
Cholesterol			0.4
kcal (% of total)	*17.2%*	*44.8%*	*34.5%*
Micronutrients			
Minerals (mg/kg)			
Calcium	5000	5000	2011
Phosphorus	3000	3000	2757
Sodium	1019	1019	7078
Potassium	3600	3600	5333
Magnesium	507	507	589
Iron	35	35	31
Zinc	30	30	25
Copper	6	6	2.6
Selenium	0.15	0.15	0.2
Vitamins (unit/kg)			
Thiamin (mg)	5	5	3.5
Riboflavin (mg)	6	6	4.4
Niacin (mg)	30	30	50.6
Pyridoxine (mg)	6	6	3.9
Folate (mg)	2	2	1.3
Vitamin B_{12} (µg)	25	25	11
Vitamin A (IU)	4000	4000	4300
Vitamin D (IU)	1000	1000	391
Vitamin E (IU)	75	75	24.6
Vitamin K (µg)	750	750	189
Choline (mg)	1027	1027	648

Abbreviations: *AIN* American Institution of Nutrition, *DIO* diet-induced obesity, *TWD* total Western diet.
Notes: Composition of the TWD was published previously [66] No data are available in NHANES for chloride, manganese, iodine, pantothenic acid, biotin, or ultra-trace minerals. Thus, levels of these components in the AIN93G diet were used in the formulation of all experimental diets.

Table S3 Primer sequences and barcodes for microbiome sequencing [1]

Barcode	Primer sequence (5'-3')	MIDD
AACCAACC	CCTACGGGRSGCAGCAG	1
AACCAAGG	CCTACGGGRSGCAGCAG	2
AACCATCG	CCTACGGGRSGCAGCAG	3
AACCATGC	CCTACGGGRSGCAGCAG	4
AACCGCAT	CCTACGGGRSGCAGCAG	5
AACCGCTA	CCTACGGGRSGCAGCAG	6
AACCGGAA	CCTACGGGRSGCAGCAG	7
AACCGGTT	CCTACGGGRSGCAGCAG	8
AACCTACG	CCTACGGGRSGCAGCAG	9
AACCTAGC	CCTACGGGRSGCAGCAG	10
AACCTTCC	CCTACGGGRSGCAGCAG	11
AACCTTGG	CCTACGGGRSGCAGCAG	12
AACGAACG	CCTACGGGRSGCAGCAG	13
AACGAAGC	CCTACGGGRSGCAGCAG	14
AACGATCC	CCTACGGGRSGCAGCAG	15
AACGATGG	CCTACGGGRSGCAGCAG	16
AACGCCAT	CCTACGGGRSGCAGCAG	17
AACGCCTA	CCTACGGGRSGCAGCAG	18
AACGCGAA	CCTACGGGRSGCAGCAG	19
AACGCGTT	CCTACGGGRSGCAGCAG	20
AACGGCAA	CCTACGGGRSGCAGCAG	21
AACGGCTT	CCTACGGGRSGCAGCAG	22
AACGTACC	CCTACGGGRSGCAGCAG	23
AACGTAGG	CCTACGGGRSGCAGCAG	24
AACGTTCG	CCTACGGGRSGCAGCAG	25
AACGTTGC	CCTACGGGRSGCAGCAG	26
AAGCAACG	CCTACGGGRSGCAGCAG	27
AAGCAAGC	CCTACGGGRSGCAGCAG	28
AAGCATCC	CCTACGGGRSGCAGCAG	29
AAGCATGG	CCTACGGGRSGCAGCAG	30
AAGCCGAA	CCTACGGGRSGCAGCAG	31
AAGCCGTT	CCTACGGGRSGCAGCAG	32
AAGCGCAA	CCTACGGGRSGCAGCAG	33
AAGCGCTT	CCTACGGGRSGCAGCAG	34
AAGCGGAT	CCTACGGGRSGCAGCAG	35
AAGCGGTA	CCTACGGGRSGCAGCAG	36
AAGCTACC	CCTACGGGRSGCAGCAG	37
AAGCTAGG	CCTACGGGRSGCAGCAG	38
AAGCTTCG	CCTACGGGRSGCAGCAG	39
AAGCTTGC	CCTACGGGRSGCAGCAG	40
AAGGAACC	CCTACGGGRSGCAGCAG	41
AAGGAAGG	CCTACGGGRSGCAGCAG	42
AAGGATCG	CCTACGGGRSGCAGCAG	43
AAGGATGC	CCTACGGGRSGCAGCAG	44
AAGGCCAA	CCTACGGGRSGCAGCAG	45
AAGGCCTT	CCTACGGGRSGCAGCAG	46
AAGGCGAT	CCTACGGGRSGCAGCAG	47
AAGGCGTA	CCTACGGGRSGCAGCAG	48
AAGGTACG	CCTACGGGRSGCAGCAG	49
AAGGTAGC	CCTACGGGRSGCAGCAG	50
AAGGTTCC	CCTACGGGRSGCAGCAG	51
AAGGTTGG	CCTACGGGRSGCAGCAG	52
AATACCGC	CCTACGGGRSGCAGCAG	53

Table S3 Primer sequences and barcodes for microbiome sequencing [1]

Barcode	Primer sequence (5’-3’)	MIDD
AATACGCC	CCTACGGGRSGCAGCAG	54
AATAGCGG	CCTACGGGRSGCAGCAG	55
AATAGGCG	CCTACGGGRSGCAGCAG	56
AATTCCGG	CCTACGGGRSGCAGCAG	57
AATTCGCG	CCTACGGGRSGCAGCAG	58
AATTCGGC	CCTACGGGRSGCAGCAG	59
AATTGCCG	CCTACGGGRSGCAGCAG	60

[1] Ion Torrent Primer A-Key CCATCTCATCCCTGCGTGTCTCCGAC; Ion Torrent Primer P-1 Key CCTCTCTATGGGCAGTCGGTGAT; key sequence TCAG; linker GAT; reverse primer sequence was ATTACCG.

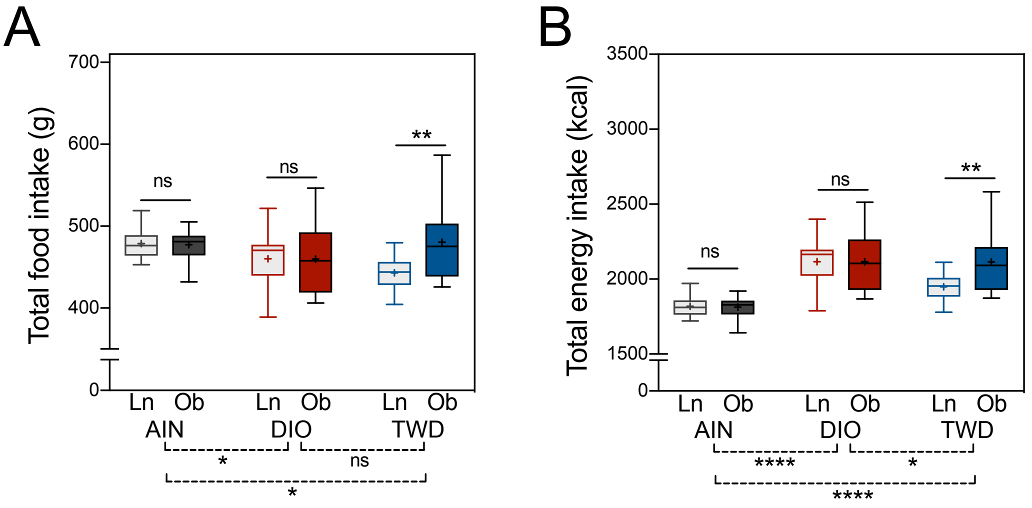

**Mixed model main effects**	***p* value**
Diet	0.0090
Body type	0.0490
Diet x body type	0.0080
Donor ID[body type]	0.7502

**Mixed model main effects**	***p* value**
Diet	<0.0001
Body type	0.0490
Diet x body type	0.0080
Donor ID[body type]	0.7502

Fig. S1 Food and energy intake. Data are presented in Tukey box-plots (box, 25th to 75th percentiles; whiskers, 1.5 IQR; +, mean) (n = 6 to 9) for total food intake (A) and total energy intake (B). The table beneath each panel shows p values for main effects of each experimental factor as determined by the mixed model analysis. Below the plot, brackets indicate results of Tukey post-hoc tests for overall effects of each diet. Within each diet group, symbols above the box-and-whisker bars indicate results of post-hoc tests comparing mice that received bacteria from lean (Ln) or obese (Ob) human donors. * p<0.05, ** p<0.01, *** p<0.001, **** p<0.0001, ns=not significant.

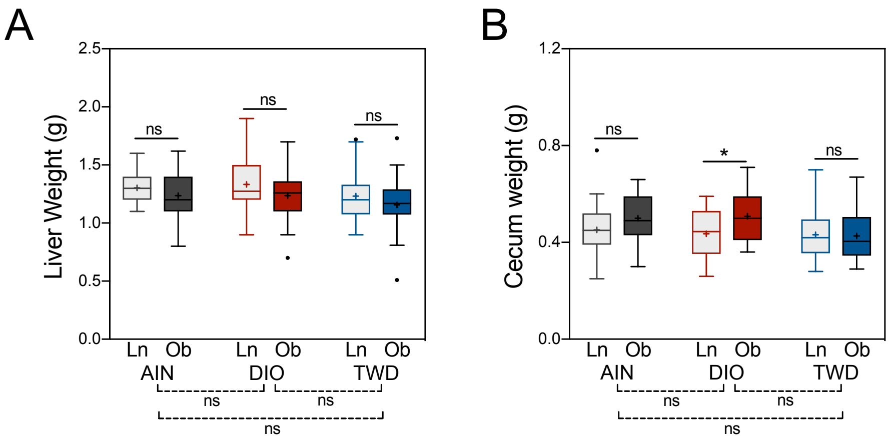

Mixed model main effects	*p* value
Diet	0.1398
Body type	0.0485
Diet x body type	0.9730
Donor ID[body type]	0.5954

Mixed model main effects	*p* value
Diet	0.1000
Body type	0.0439
Diet x body type	0.2228
Donor ID[body type]	0.1740

Fig. S2 Liver and cecum weights. Data for liver weight (A) and cecum weight (B) are shown in Tukey box-plots (box, 25th to 75th percentiles; whiskers, 1.5 IQR; +, mean) (n = 6 to 9). The tables below each panel show p values for main effects of each experimental factor as determined by the mixed model analysis. Below each plot, brackets indicate results of Tukey post-hoc tests for overall effects of each diet. Within each diet group, symbols above the box-and-whisker bars indicate results of post-hoc tests comparing mice that received bacteria from lean (Ln) or obese (Ob) human donors. * p<0.05, ** p<0.01, *** p<0.001, **** p<0.0001, ns=not significant.

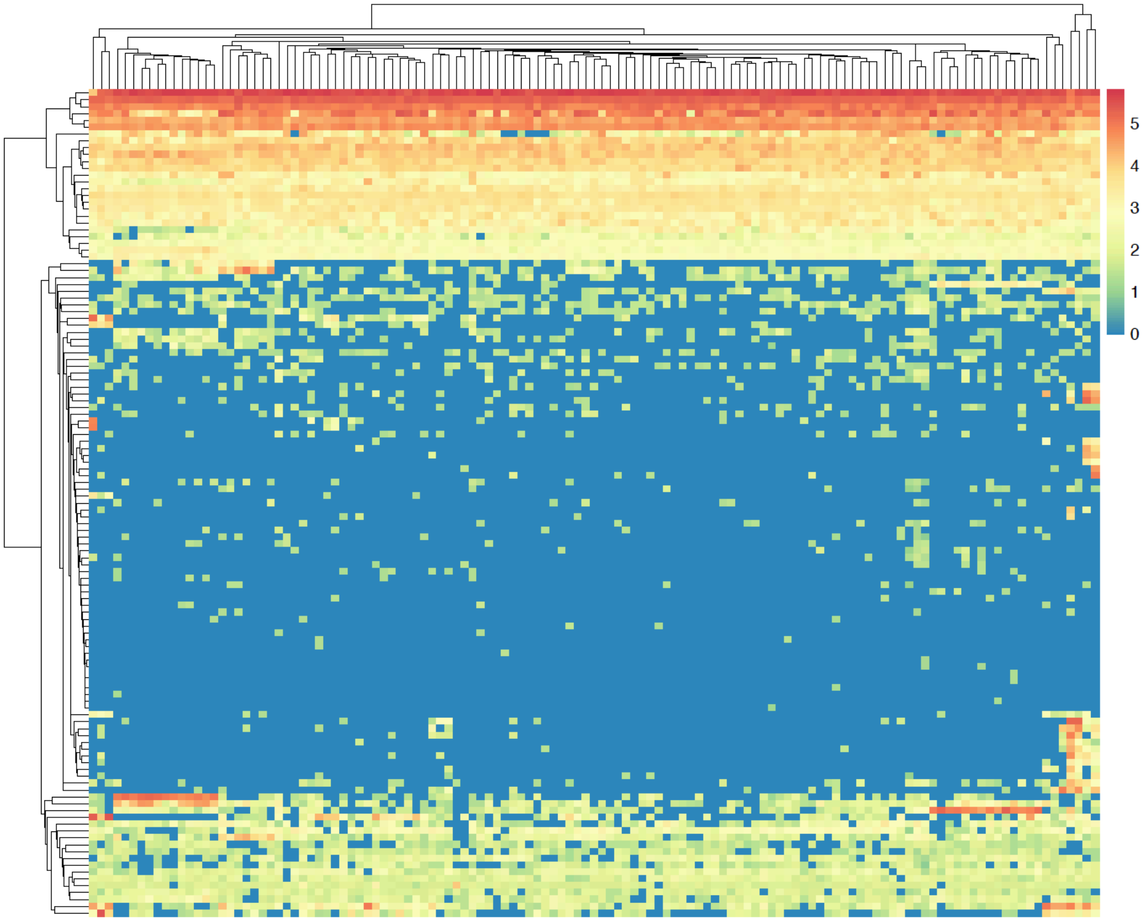

Fig. S3 Heatmap depicting bidirectional, unsupervised hierarchical cluster analysis of initial mouse fecal microbiomes. Values shown are the normalized reads for all mapped OTUs in the initial fecal mouse microbiomes, prior to antibiotic or diet treatments. Clustering was performed using Euclidian distance with average linkage. Heatmap color scale indicates relative abundance calculated as the $\log_{10}$ reads per million for better visualization of lower abundance OTUs.

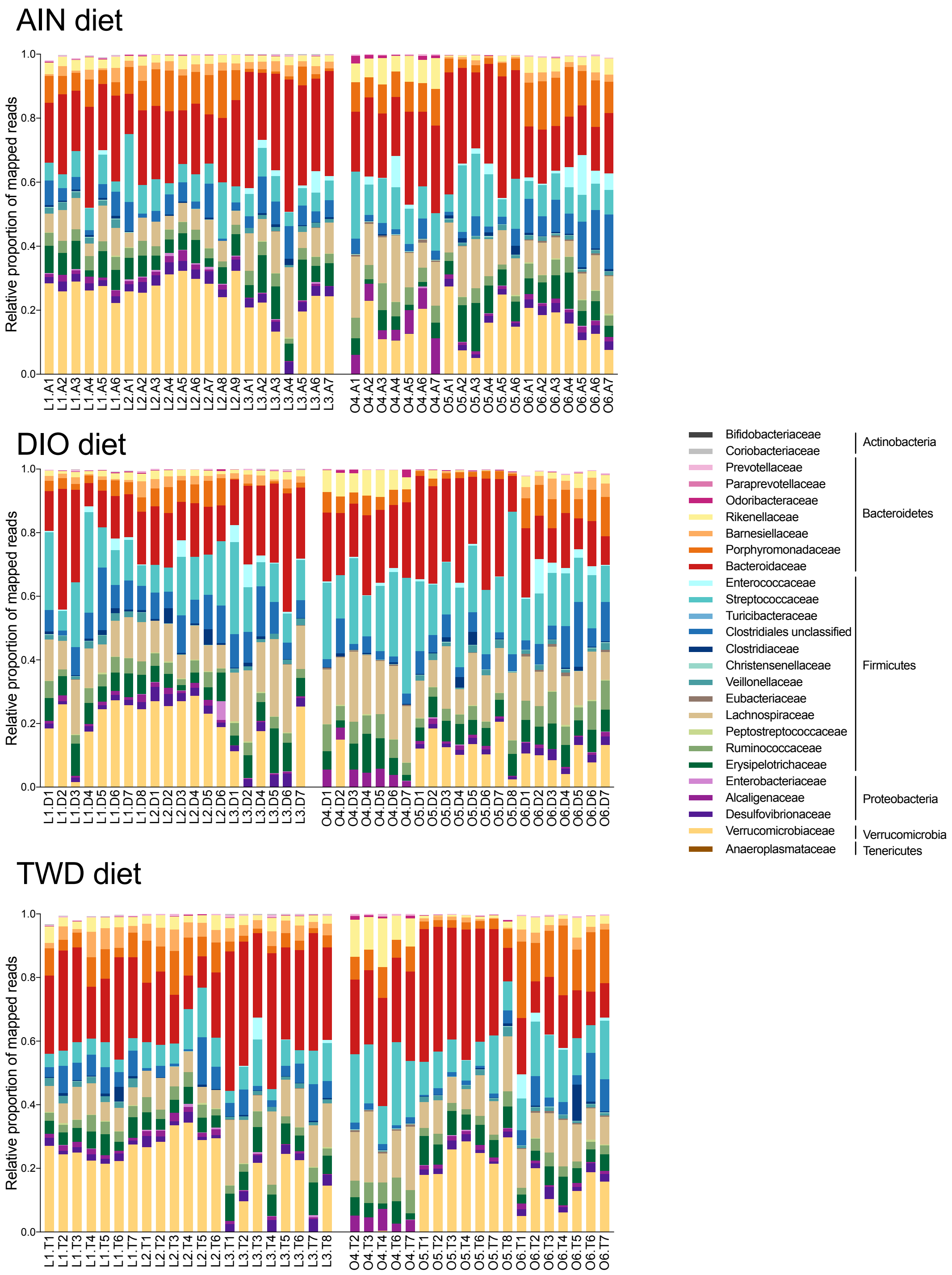

Fig. S4 Classification of human or mouse fecal bacteria by family for individual mice post fecal microbiome transfer. Data shown are the relative abundance of bacteria to annotated taxa (phylum and family) for individual mice for each experimental diet group. Mouse identification codes that begin with *L* indicate recipients of FMT from lean human donors, and codes that begin with *O* indicate recipients of FMT from obese human donors.

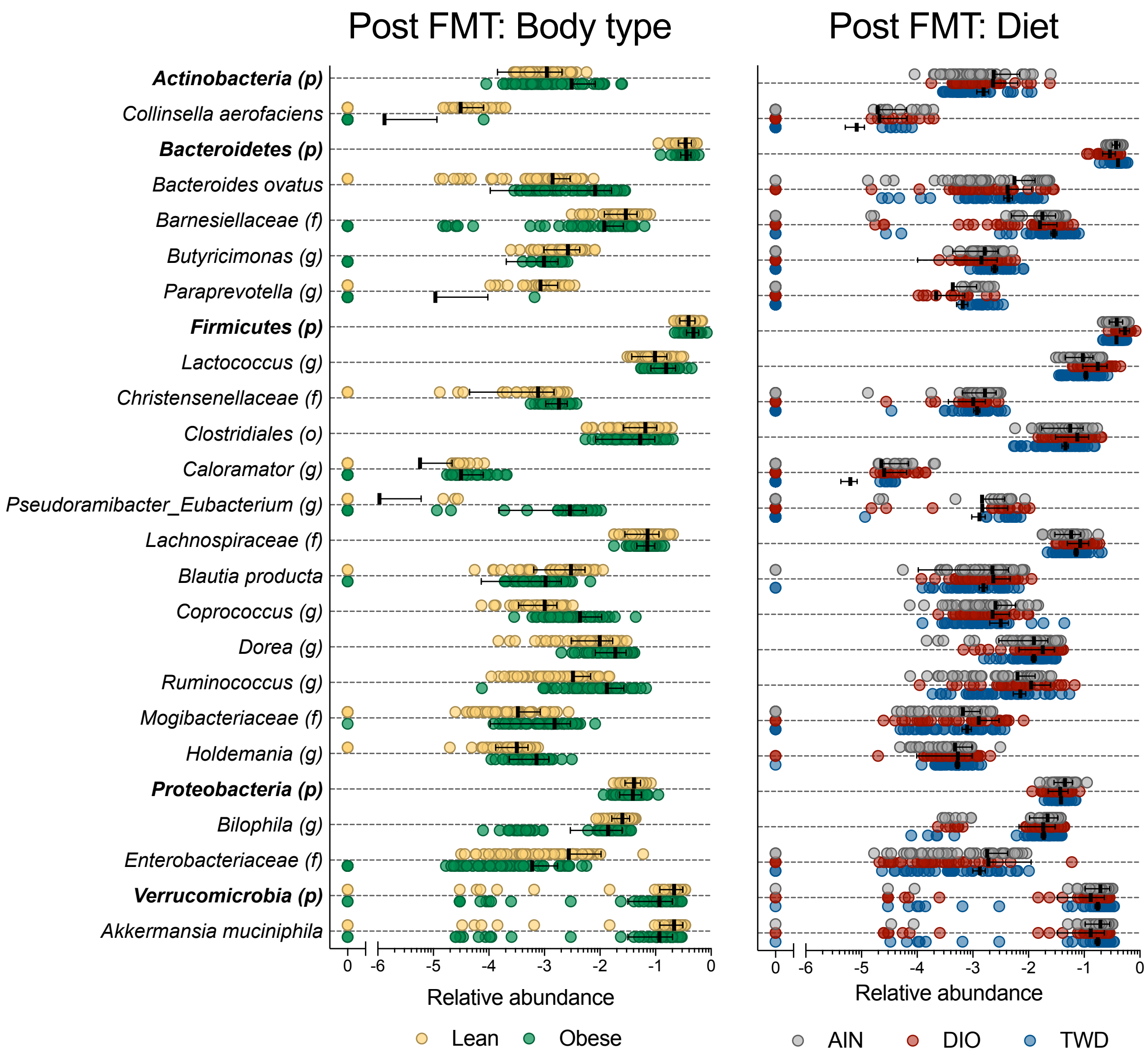

Fig. S5 Relative abundance of selected bacteria taxa by main factors, body type and experimental diet, post fecal microbiome transfer. Data shown are individual relative abundance values for each mouse, with the black bar and whiskers representing the mean ± SEM on a $\log_{10}$ scale to facilitate visualization of less abundant species. Bold titles indicate the major represented phyla, with members of interest listed below.

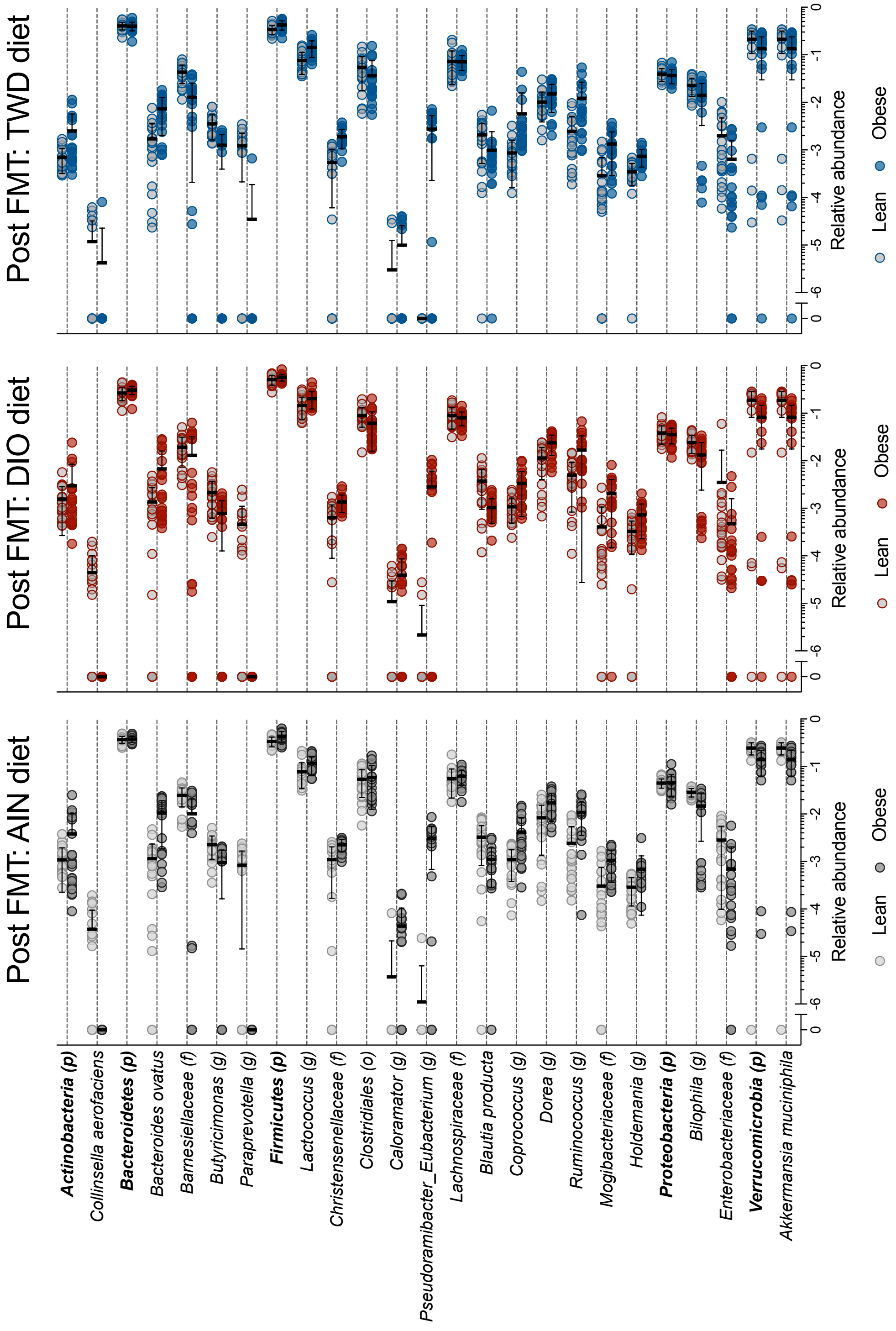

Fig. S6 Relative abundance of selected bacteria taxa in lean or obese recipient mice stratified by experimental diet post fecal microbiome transfer. Data shown are individual relative abundance values for each mouse, with the black bar and whiskers representing the mean ± SEM on a $\log_{10}$ scale to facilitate visualization of less abundant species. Bold titles indicate the major represented phyla, with members of interest listed below.

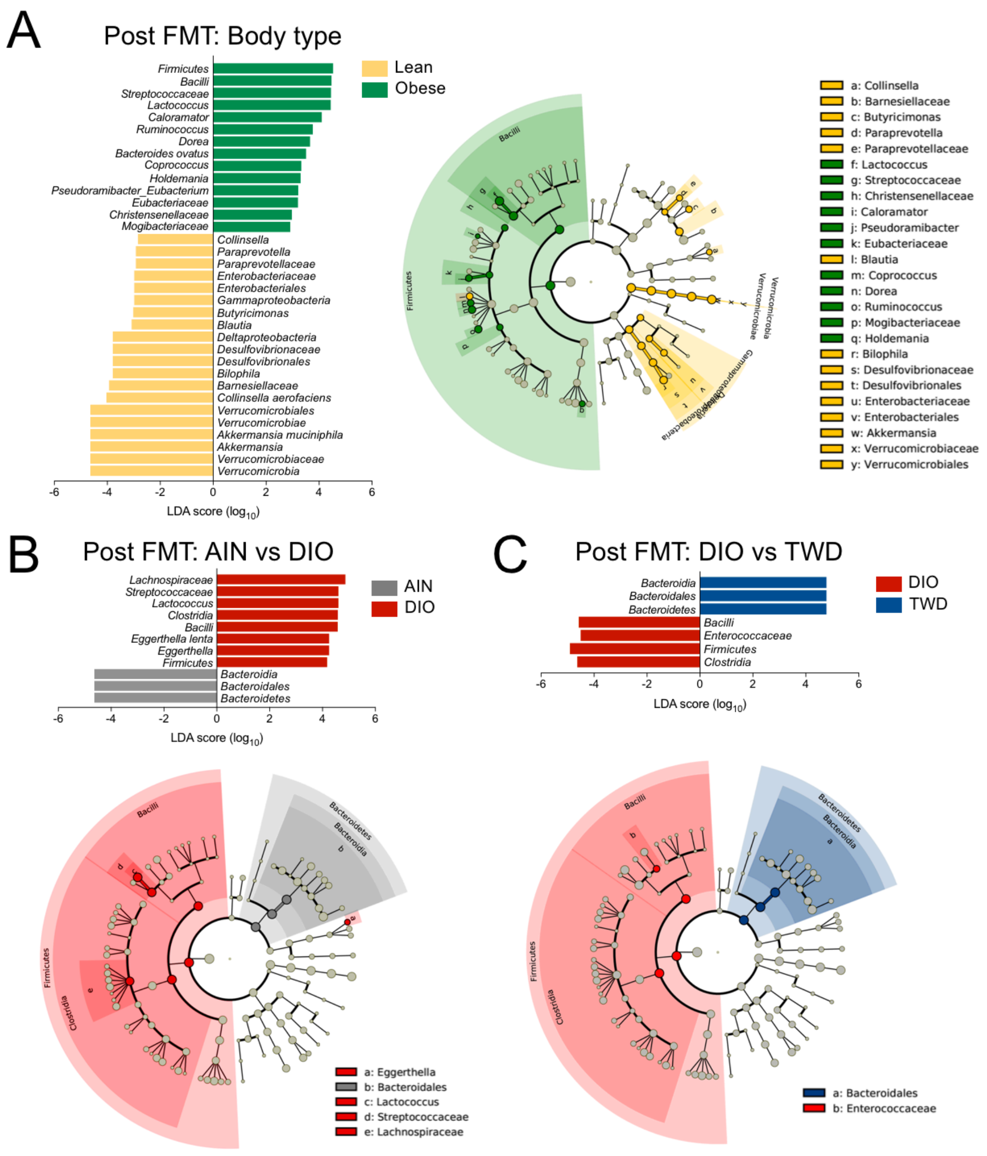

Fig. S7 Discriminating taxa for recipient mouse microbiomes post fecal transfer. LEfSe analyses were performed using relative abundance data averaged by human donor at the highest classification level taxonomy available. Data shown are the log_{10} linear discriminant analysis (LDA) scores following LEfSe analyses and the hierarch of discriminating taxa visualized as cladograms for the following class comparisons: (A) all lean donor recipient mice compared to all obese donor recipient mice, (B) all mice fed AIN diet compared to those fed DIO diet, and (C) all mice fed DIO diet compared to those fed TWD. (No discriminating features were identified for all mice fed AIN compared to those fed TWD.) The structure of these comparisons was designed to mimic tests for main effects of body type and diet (including diet pairwise tests), as was done for other study parameters using the linear mixed model. Relative abundance data of selected taxa of interest are shown in Fig. S4.

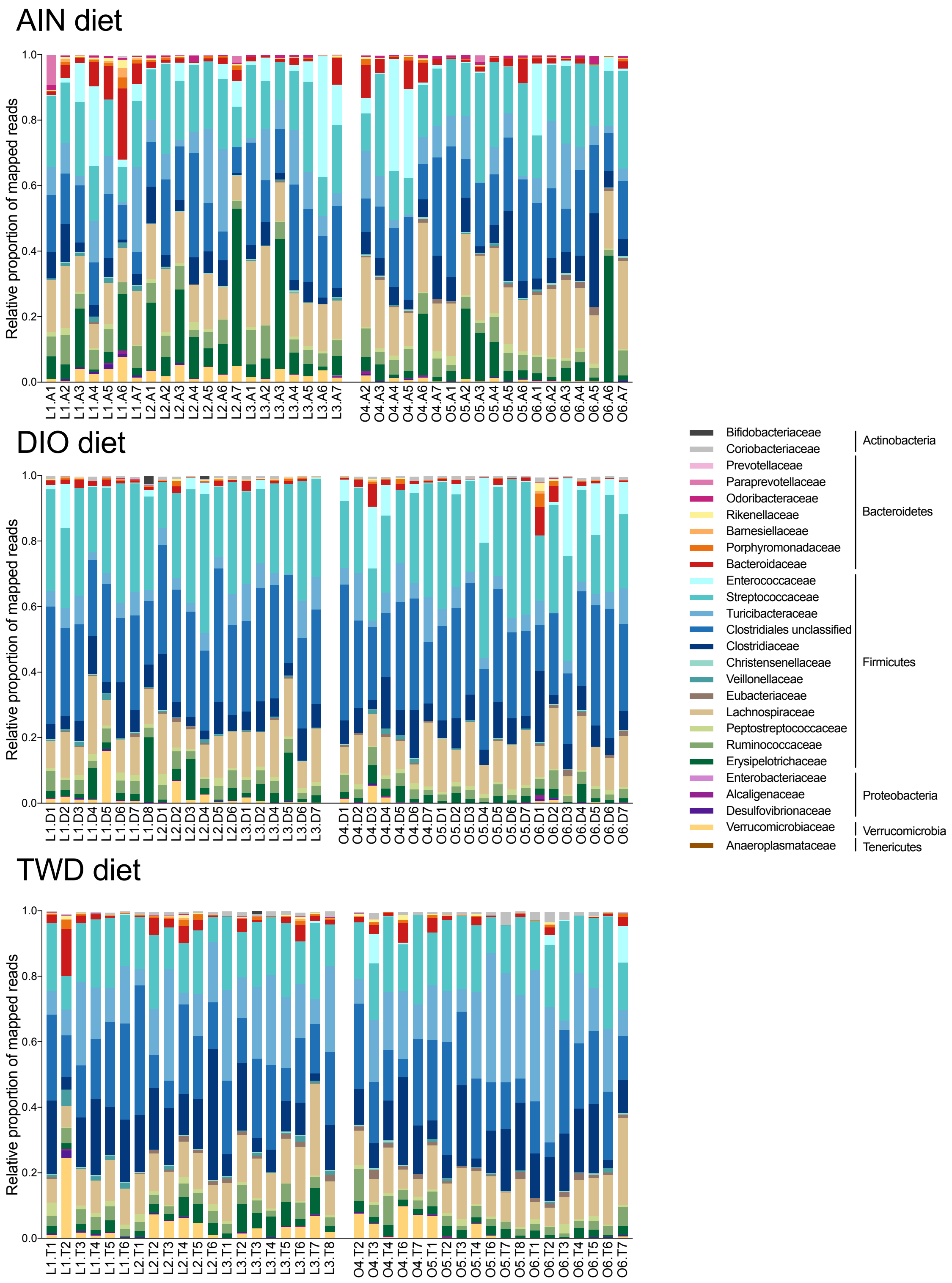

Fig. S8 Classification of human or mouse fecal bacteria by family for individual mice at necropsy. Data shown are the relative abundance of bacteria to annotated taxa (phylum and family) for individual mice for each experimental diet group. Mouse identification codes that begin with *L* indicate recipients of FMT from lean human donors, and codes that begin with *O* indicate recipients of FMT from obese human donors.

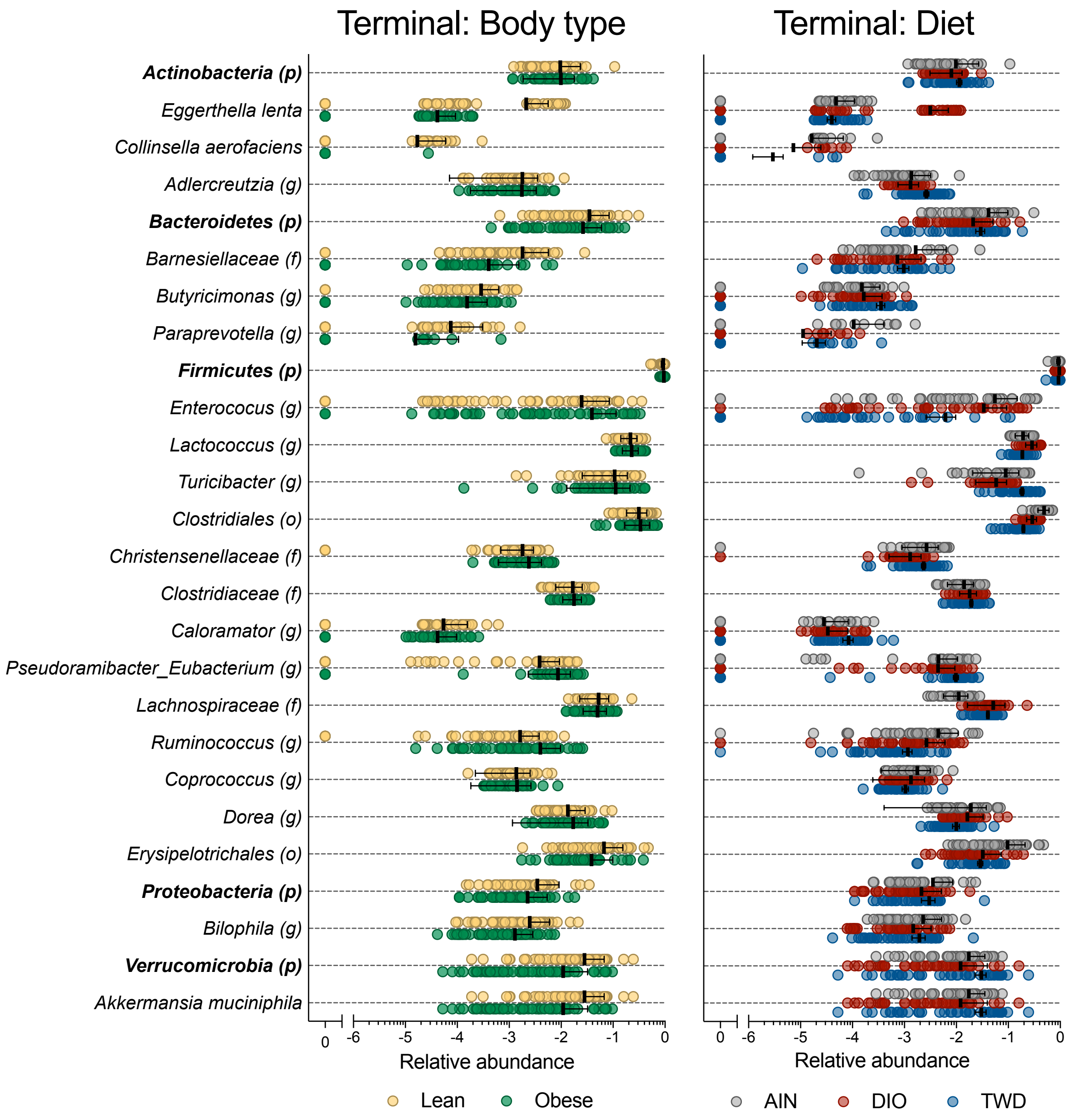

Fig. S9 Relative abundance of selected bacteria taxa by main factors, body type and experimental diet, at necropsy. Data shown are individual relative abundance values for each mouse, with the black bar and whiskers representing the mean ± SEM on a $\log_{10}$ scale to facilitate visualization of less abundant species. Bold titles indicate the major represented phyla, with members of interest listed below.

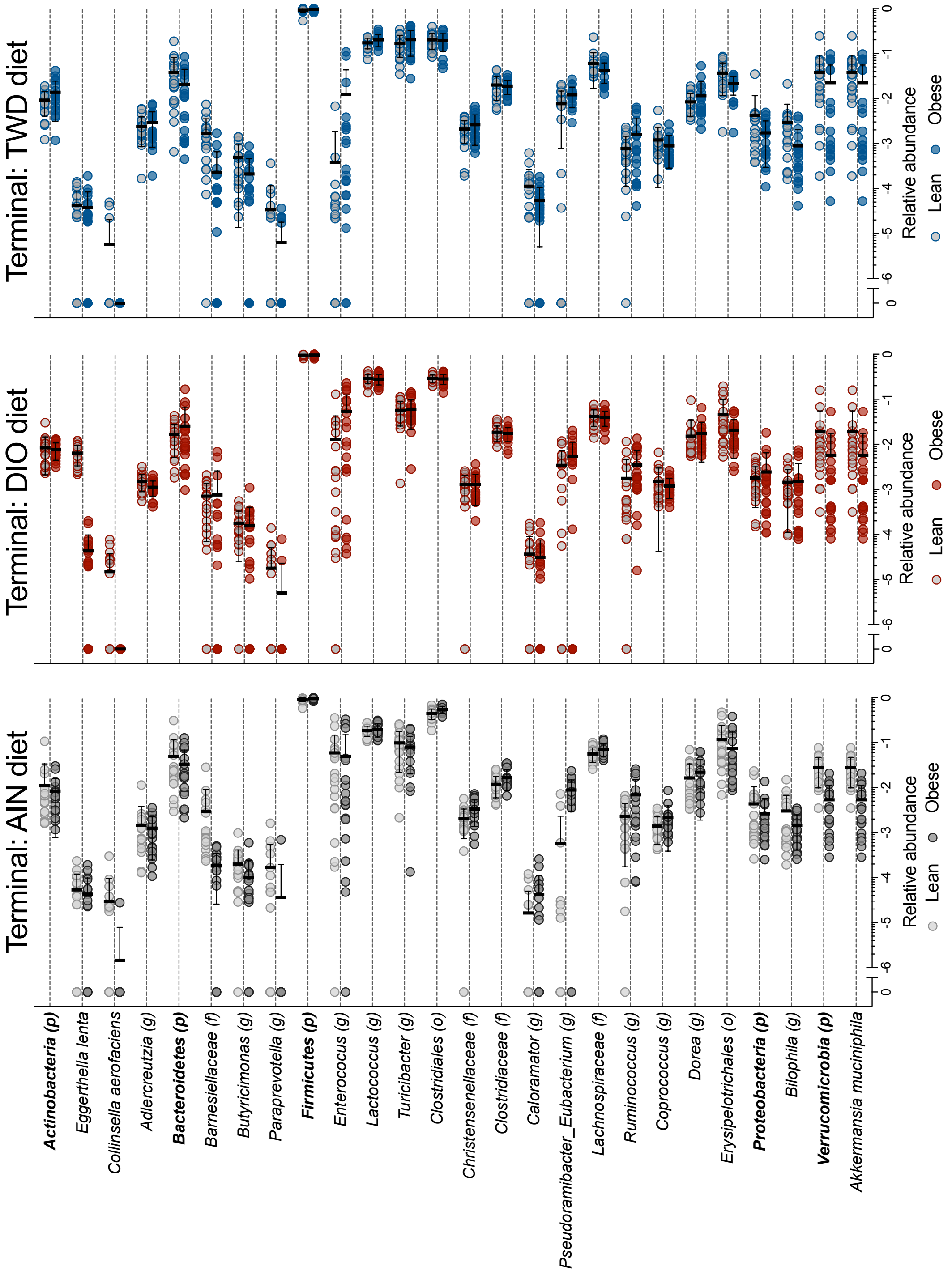

Fig. S10 Relative abundance of selected bacteria taxa in lean or obese recipient mice stratified by experimental diet at necropsy. Data shown are individual relative abundance values for each mouse, with the black bar and whiskers representing the mean ± SEM on a $\log_{10}$ scale to facilitate visualization of less abundant species. Bold titles indicate the major represented phyla, with members of interest listed below.

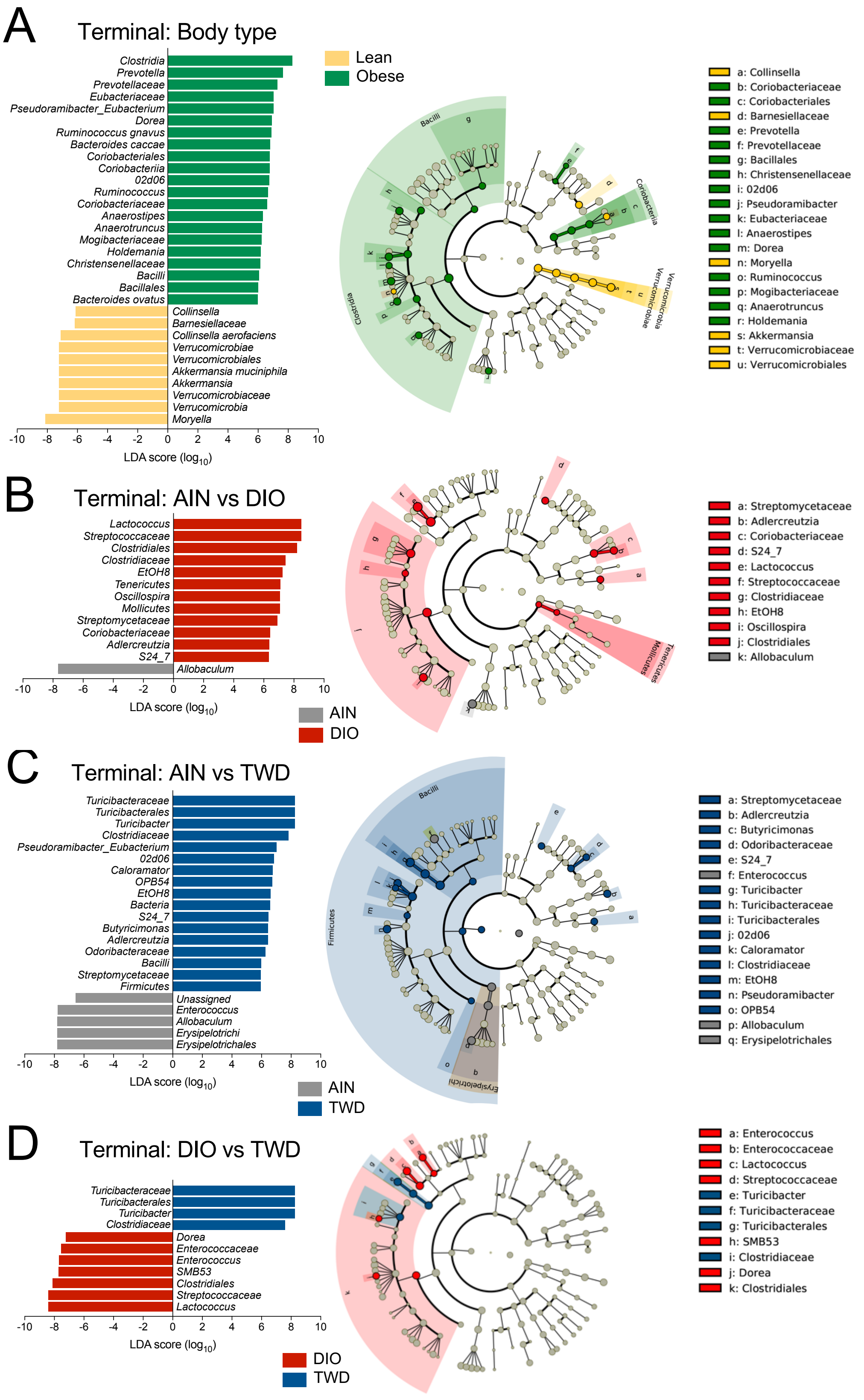
A
Terminal: Body type
Lean
Obese
Clostridia
Prevotella
Prevotellaceae
Eubacteriaceae
Pseudoramibacter_Eubacterium
Dorea
Ruminococcus gnavus
Bacteroides caccae
Coriobacteriales
Coriobacteriia
02d06
Ruminococcus
Coriobacteriaceae
Anaerostipes
Anaerotruncus
Mogibacteriaceae
Holdemania
Christensenellaceae
Bacilli
Bacillales
Bacteroides ovatus
Collinsella
Barnesiellaceae
Collinsella aerofaciens
Verrucomicrobiae
Verrucomicrobiales
Akkermansia muciniphila
Akkermansia
Verrucomicrobiaceae
Verrucomicrobia
Moryella
-10 -8 -6 -4 -2 0 2 4 6 8 10
LDA score (log10)
Bacilli
Clostridia
Coriobacteriia
Verrucomicrobia
Verrucomicrobiae
a: Collinsella
b: Coriobacteriaceae
c: Coriobacteriales
d: Barnesiellaceae
e: Prevotella
f: Prevotellaceae
g: Bacillales
h: Christensenellaceae
i: 02d06
j: Pseudoramibacter
k: Eubacteriaceae
l: Anaerostipes
m: Dorea
n: Moryella
o: Ruminococcus
p: Mogibacteriaceae
q: Anaerotruncus
r: Holdemania
s: Akkermansia
t: Verrucomicrobiaceae
u: Verrucomicrobiales
B
Terminal: AIN vs DIO
Lactococcus
Streptococcaceae
Clostridiales
Clostridiaceae
EtOH8
Tenericutes
Oscillospira
Mollicutes
Streptomycetaceae
Coriobacteriaceae
Adlercreutzia
S24_7
Allobaculum
-10 -8 -6 -4 -2 0 2 4 6 8 10
LDA score (log10)
AIN
DIO
Tenericutes
Mollicutes
a: Streptomycetaceae
b: Adlercreutzia
c: Coriobacteriaceae
d: S24_7
e: Lactococcus
f: Streptococcaceae
g: Clostridiaceae
h: EtOH8
i: Oscillospira
j: Clostridiales
k: Allobaculum
C
Terminal: AIN vs TWD
Turicibacteraceae
Turicibacterales
Turicibacter
Clostridiaceae
Pseudoramibacter_Eubacterium
02d06
Caloramator
OPB54
EtOH8
Bacteria
S24_7
Butyricimonas
Adlercreutzia
Odoribacteraceae
Bacilli
Streptomycetaceae
Firmicutes
Unassigned
Enterococcus
Allobaculum
Erysipelotrichi
Erysipelotrichales
-10 -8 -6 -4 -2 0 2 4 6 8 10
LDA score (log10)
AIN
TWD
Bacilli
Firmicutes
Erysipelotrichi
a: Streptomycetaceae
b: Adlercreutzia
c: Butyricimonas
d: Odoribacteraceae
e: S24_7
f: Enterococcus
g: Turicibacter
h: Turicibacteraceae
i: Turicibacterales
j: 02d06
k: Caloramator
l: Clostridiaceae
m: EtOH8
n: Pseudoramibacter
o: OPB54
p: Allobaculum
q: Erysipelotrichales
D
Terminal: DIO vs TWD
Turicibacteraceae
Turicibacterales
Turicibacter
Clostridiaceae
Dorea
Enterococcaceae
Enterococcus
SMB53
Clostridiales
Streptococcaceae
Lactococcus
-10 -8 -6 -4 -2 0 2 4 6 8 10
LDA score (log10)
DIO
TWD
a: Enterococcus
b: Enterococcaceae
c: Lactococcus
d: Streptococcaceae
e: Turicibacter
f: Turicibacteraceae
g: Turicibacterales
h: SMB53
i: Clostridiaceae
j: Dorea
k: Clostridiales

Fig. S11 Discriminating taxa for recipient mouse microbiomes at necropsy. LEfSe analyses were performed using relative abundance data averaged by human donor at the highest classification level taxonomy available. Data shown are the $\log_{10}$ linear discriminant analysis (LDA) scores following LEfSe analyses and the hierarch of discriminating taxa visualized as cladograms for the following class comparisons: (A) all lean donor recipient mice compared to all obese donor recipient mice, (B) all mice fed AIN diet compared to those fed DIO diet, and (C) all mice fed DIO diet compared to those fed TWD. (No discriminating features were identified for all mice fed AIN compared to those fed TWD.) The structure of these comparisons was designed to mimic tests for main effects of body type and diet (including diet pairwise tests), as was done for other study parameters using the linear mixed model. Relative abundance data of selected taxa of interest are shown in Fig. S6.

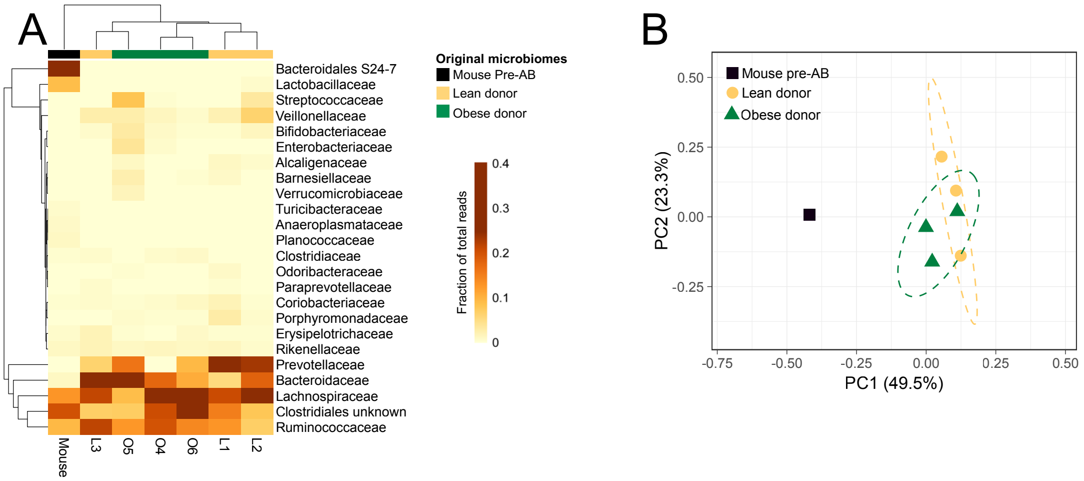

Fig. S12 Hierarchical clustering and principal components analysis of original mouse and human donor microbiomes. (A) Unsupervised, bidirectional hierarchical cluster analysis of taxonomy relative abundance data for bacteria families comprising a least 1% of the fecal microbiome from all mice prior to antibiotic treatment (pre-AB) and from each of the six human donors (lean: L1, L2, L3; obese, O4, O5, O6). Clustering was performed using Euclidian distance with average linkage. Heatmap color scale indicates relative abundance as fraction of total reads. (B) PCA of family-level taxonomy data plotted according to the first two principal components. Variation attributed to each component is shown. Dashed lines represent the 95% confidence ellipse.

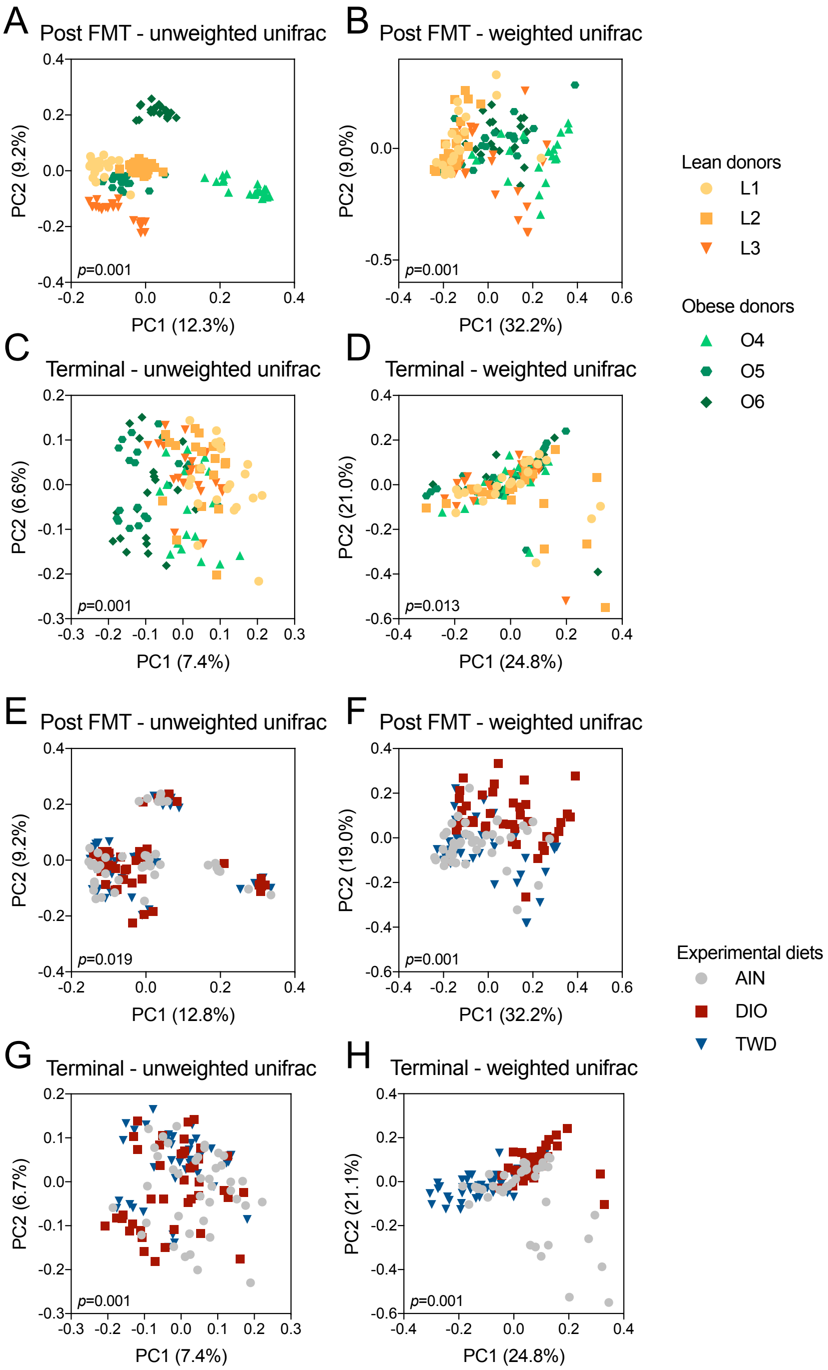

Fig. S13 Beta diversity principal coordinates plots for weighted and unweighted unifrac distances for post FMT and terminal recipient mouse microbiomes. Data shown are the first two principal components of unweighted or weighted unifrac distances for post FMT and terminal fecal microbiomes categorized by human donor body type (A-D) or experimental diet (E-H). Variation attributed to each coordinate is shown. Permanova *p* values are shown for each plot.
